# Combined immunoscore and pan-immune inflammation value associated with pathological response and survival outcomes in esophageal squamous cell carcinoma receiving neoadjuvant immunotherapy

**DOI:** 10.1097/JS9.0000000000003559

**Published:** 2025-10-07

**Authors:** Jiang-shan Huang, Qi-hong Zhong, Ye-qin Zhang, Huai-yuan Zhang, Fei-long Guo, Jing-yu Wu, Sui Chen, Wen-wei Lin, Zhen-yang Zhang, Jiang-bo Lin

**Affiliations:** a Department of Thoracic Surgery, Fujian Medical University Union Hospital, Fuzhou, China; b Key Laboratory of Cardio-Thoracic Surgery (Fujian Medical University), Fujian Province University, Fuzhou, China; c National Key Clinical Specialty of Thoracic Surgery, Fuzhou, China; d Clinical Research Center for Thoracic Tumors of Fujian Province, Fuzhou, China

**Keywords:** esophageal squamous cell carcinoma, immunoscore, neoadjuvant chemoimmunotherapy, pan-immune inflammation value, predictive biomarker

## Abstract

**Objective:**

To evaluate the efficacy of combined tumor immunoscore (IS) and pan-immune inflammation value (PIV) in assessing pathological response and survival following neoadjuvant chemoimmunotherapy (NACI) in locally advanced esophageal squamous cell carcinoma (ESCC).

**Methods:**

A retrospective cohort of 226 ESCC patients undergoing NACI followed by surgery was analyzed. Local immune status was assessed via IS calculated from CD3⁺/CD8⁺ T-cell densities on immunohistochemical stained tumor sections. Systemic inflammation was measured by peripheral blood PIV. Patients were stratified by median values. The association of IS, PIV alone, and their combination for major pathological response (TRG 0–1) and overall survival (OS) was evaluated.

**Results:**

The pathological response rate was significantly higher in the high IS group versus the low IS group (54.3% vs. 38.6%; *P* = 0.023) and in the low PIV group p versus the high PIV group (67.3% vs. 21.2%; *P* < 0.001). In the combined model, the low IS + high PIV group (Group D) exhibited the lowest pathological response rate (32.0%) and a significantly increased risk of death compared with the optimal group [high IS + low PIV; Group A; hazard ratios = 2.85; 95% confidence interval (CI): 1.70–4.77; *P* < 0.001]. The dual-parameter IS + PIV model demonstrated superior discriminatory performance: area under the curve (AUC) = 0.78 (95% CI: 0.73–0.84) for discriminating pathological response and AUC = 0.82 (95% CI: 0.75–0.88) for predicting 36-month OS, significantly outperforming single parameters and clinical staging.

**Conclusion:**

The IS + PIV dual-parameter model, by integrating local immune activity and systemic inflammatory status, accurately identifies NACI beneficiaries (high IS + low PIV) and high-risk patients (low IS + high PIV), providing an efficient prognostic tool for personalized treatment strategies.

**HIGHLIGHTS**

- **Novel dual-parameter model**: Combined local immunoscore (IS) and systemic pan-immune inflammation value (PIV) predicts neoadjuvant chemoimmunotherapy response in esophageal squamous cell carcinoma.
- **Superior predictive power**: IS + PIV achieved area under the curve (AUC) = 0.78 (pathological response) and AUC = 0.82 [36-month overall survival (OS)].
- **Precise risk stratification**: *Optimal group* (high IS + low PIV): 48.8% pathological response, best OS; *high-risk group* (low IS + high PIV): 32.0% pathological response, HR = 2.85 for death.
- **Clinically feasible**: Uses routine immunohistochemical (CD3/CD8) and CBC parameters, cost-effective for clinical use.

## Background

Neoadjuvant chemoimmunotherapy (NACI) has emerged as a pivotal therapeutic strategy for locally advanced esophageal squamous cell carcinoma (ESCC), significantly enhancing pathological response rates and showing potential for improved patient survival^[1]^. However, not all patients derive equivalent clinical benefit, and effective biomarkers for precise identification of potential responders remain lacking. The efficacy of NACI is highly dependent on activated anti-tumor immune responses. Both the status of the local tumor immune microenvironment – specifically the infiltration density and spatial distribution of cytotoxic T lymphocytes (e.g., CD8^+^ T cells) and total T lymphocytes (CD3^+^) – and systemic immune-inflammatory activity [reflected by peripheral blood biomarkers such as cytokines or cellular ratios, including the pan-immune inflammation value (PIV) that integrates neutrophils, monocytes, platelets, and lymphocytes] have been demonstrated to correlate significantly with immunotherapy response and prognosis^[2,3]^.

Although the immunoscore (IS) and PIV have demonstrated value as independent prognostic markers in select studies, current evidence remains largely confined to single-dimensional analyses. Tumor immune responses constitute a complex interplay between local and systemic processes; thus, isolated assessment of either local immune infiltration or systemic inflammation may inadequately capture a patient’s comprehensive immunologic status^[4]^. To address this gap, we aim to investigate the combined role of postoperative tumor tissue IS and preoperative peripheral blood PIV. We will evaluate whether this integrated analysis surpasses individual biomarkers in assessing both pathological response (assessed by tumor regression grading, TRG) and predicting long-term survival outcomes (overall survival, OS) in ESCC patients undergoing curative esophagectomy following NACI. This approach seeks to identify a more reliable biomarker composite for optimizing patient selection and prognostication in clinical practice.

## Materials and methods

### Study population

**Figure 1.**
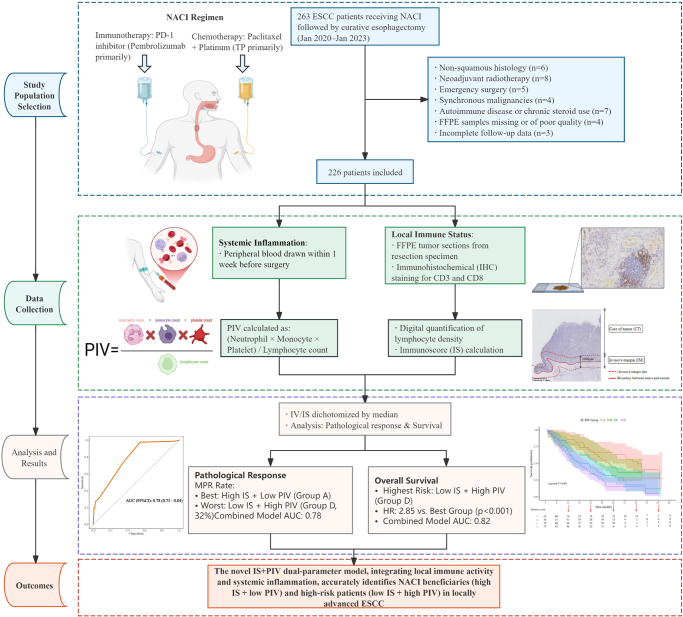
This diagram illustrates the process of patient enrollment, data analysis, and the derivation of results and conclusions.

A retrospective cohort of 263 consecutive patients with ESCC who underwent curative esophagectomy following NACI was enrolled between January 2020 and January 2023. **Inclusion criteria** comprised (1) histologically confirmed ESCC by pretreatment biopsy; (2) clinical stage II–IVA (8th edition UICC/AJCC TNM staging); (3) completion of curative esophagectomy; (4) NACI regimen including PD-1 inhibitors [nivolumab (200 mg, Q3W) or pembrolizumab (200 mg, Q3W)] combined with platinum-based chemotherapy [primally paclitaxel-platinum (TP)]; (5) complete medical records and follow-up data. **Exclusion criteria** were (1) adenocarcinoma or non-squamous histology; (2) non-chemotherapy/immunotherapy neoadjuvant modalities (e.g., radiotherapy); (3) Emergency surgery due to complications (e.g., perforation/hemorrhage); (4) synchronous malignancies; (5) autoimmune diseases or systemic corticosteroid use (>1 month); (6) formalin-fixed paraffin-embedded (FFPE) samples missing or of poor quality; (7) incomplete follow-up data. According to the inclusion and exclusion criteria, a total of 226 patients were ultimately included in the analysis (Fig. 1). FFPE tumor specimens from surgical resection were collected for immunohistochemistry (IHC).

This study complied with the Declaration of Helsinki and was approved by the Institutional Review Board, with written informed consent obtained from all participants. Furthermore, the work has been reported in line with the STROCSS criteria^[5]^.

### CD3 and CD8 immunohistochemical staining

FFPE tissue sections (4 μm) were processed as follows: Dewaxed in xylene, rehydrated through graded ethanol, and subjected to antigen retrieval in pH 6.0 buffer (110°C, 20 minutes, pressure cooker). Endogenous peroxidase was blocked with 0.3% H_2_O_2_/methanol (20 minutes), and nonspecific binding sites were inhibited with normal horse serum. Sections were incubated overnight at 4°C with mouse monoclonal antibodies against CD3 (clone C8/144B, Dako, 1:500) and CD8 (clone C8/144B, Dako, 1:500), followed by 20-minute incubation with biotinylated secondary antibody at room temperature. Immune complexes were visualized using Vectastain ABC kit (20 minutes) and DAB (3,3’-Diaminobenzidin) substrate (2.5 minutes), counterstained with hematoxylin, and mounted. Human tonsil tissue served as a positive control.

### Tumor immune microenvironment evaluation

**Figure 2.**
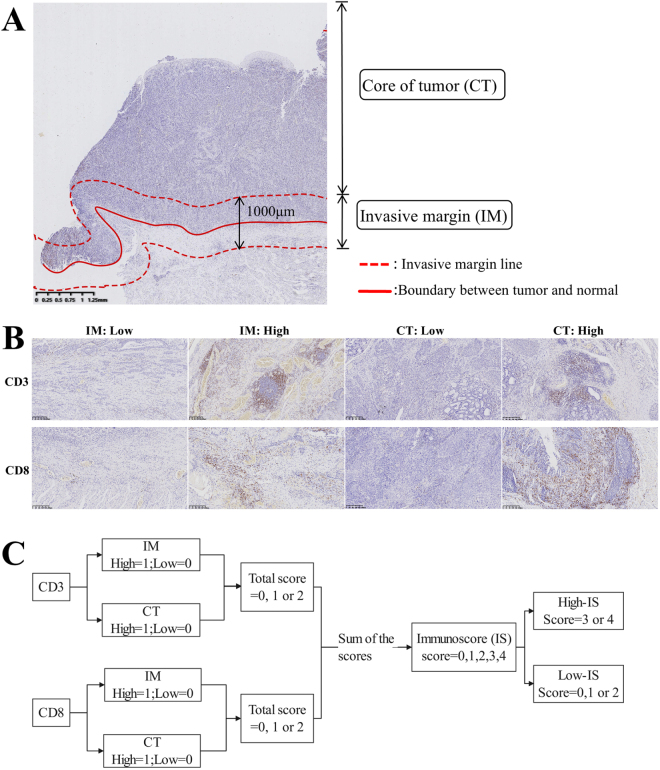
Immunostaining of CD3^+^ and CD8^+^ cells for immune score evaluation. (A) Representative CD8 immunostaining section of (Esophageal Carcinoma) EC resection specimens showing typical tumor regions, including the central tumor (CT) and invasive margin (IM) (original magnification: 20×). (B) Control and representative slides demonstrating low or high density of CD3^+^ and CD8^+^ lymphocytes in CT or IM. (C) Schematic diagram illustrating the calculation model of the immunoscore (IS).

The IS system was rigorously implemented according to published criteria^[6,7]^. The invasive margin (IM) zone was defined as a 500-μm band extending inward and outward from the tumor-normal interface, while the central tumor (CT) zone comprised all tumor areas interior to the IM. Within CT and IM regions, five lymphocyte density hotspots (500 × 500 μm fields) were selected. CD3^+^ and CD8^+^ lymphocytes were quantified using Keyence BZ-X710 microscopy (200× magnification) with BZ-H3C automated counting software. IS calculation followed a three-step protocol: first, marker-specific densities in CT/IM zones were assigned 1 point if exceeding cohort median values or 0 points otherwise; subsequently, scores from both zones were summed to yield CD3 (0–2) and CD8 scores (0–2); finally, composite IS (0–4) categorized patients into IS-low (0–2) and IS-high (3–4) groups. For IM density analysis, total CD3^+^/CD8^+^ cell counts were divided by IM area (mm^2^) calculated via BZ-H3C software. All assessments were independently performed by two blinded observers, with discrepancies resolved by consensus and final validation by a senior pathologist (Fig. 2).

### PIV assessment

The systemic immune-inflammatory status before surgery was assessed using the PIV. Peripheral blood samples for PIV calculation were collected after patients had completed NACI and were deemed eligible for surgery, within 1 week prior to curative esophagectomy. PIV was computed as (neutrophil count × monocyte count × platelet count)/lymphocyte count. Patients were stratified into high-PIV (>cohort median) and low-PIV (≤median) groups based on this value.

All blood samples were processed in accordance with standard clinical protocols: venous blood was collected in Ethylenediaminetetraacetic acid (EDTA) anticoagulant tubes and analyzed within 2 hours of collection using a uniform automated hematology analyzer (Mindray BC-7500; Mindray Bio-Medical Electronics, Shenzhen, China) in the central laboratory. To minimize inter-assay variation, all testing was performed in the same clinical laboratory by designated personnel following identical standardized procedures.

### Postoperative pathological response and follow-up

Pathological response was assessed using the modified Ryan TRG: TRG 0 (complete response), TRG 1 (moderate response), TRG 2 (minimal response), and TRG 3 (no response). Patients were classified as responders (TRG 0–1) or non-responders (TRG 2–3). OS served as the primary endpoint. Survival status was verified through scheduled telephone follow-ups by a dedicated research team, with final confirmation of vital status and death dates conducted at the analysis cutoff date.

### Statistical analysis

Data were described according to distributional characteristics: normally distributed continuous variables as mean ± standard deviation (mean ± SD), skewed continuous variables as median (interquartile range) [M (IQR)], and categorical variables as frequency and percentage (*n*, %). Intergroup comparisons utilized one-way analysis of variance for normally distributed continuous variables, with chi-square tests or chi-square trend tests for categorical variables. Univariable and multivariable logistic regression models evaluated associations between variables and pathological response, generating odds ratios (ORs) with 95% confidence intervals (CIs). Similarly, univariable and multivariable Cox proportional hazards regression models analyzed associations with survival outcomes, reporting hazard ratios (HRs) and 95% CIs. Survival curves were constructed using the Kaplan–Meier method, with between-group differences assessed by log-rank tests. Interaction analyses examined combined effects of IS and PIV. The discriminatory performance for pathological response and predictive performance for survival outcomes was evaluated for clinical stage, IS alone, PIV alone, and IS combined with PIV (IS + PIV). Statistical analyses were performed using SPSS 26.0, GraphPad Prism 10.3, R 4.2.3, and Zstats v0.90 (http://www.medsta.cn/software).

A post-hoc power calculation was performed for the key survival comparison between the high-IS + low-PIV group (Group A, *n* = 43) and the low-IS + high-PIV group (Group D, *n* = 75), using the observed hazard ratio of 2.85. With a two-sided alpha level of 0.05, the analysis yielded a statistical power of 99.3%, confirming that the sample size was more than sufficient to detect this clinically significant difference in OS.

## Results

### Baseline characteristics of the study population

The final cohort comprised 226 ESCC patients undergoing curative esophagectomy after NACI. Median age was 60 years (IQR: 54.00–65.75), with male predominance (79.65%). Most patients were American Society of Anesthesiologists Physical Status Classification (ASA) class II (88.05%), had mid-esophageal tumors (56.64%), and presented with clinical stage III disease (80.97%). The TP regimen (paclitaxel-platinum) constituted the primary chemotherapy backbone (88.50%), and pembrolizumab was the most frequent PD-1 inhibitor (55.31%). Patients stratified by IS into low-IS (*n* = 145) and high-IS (*n* = 81) groups showed comparable demographics, though clinical stage distribution differed significantly (*P* = 0.028). PIV dichotomization (low-PIV: *n* = 113; high-PIV: *n* = 113) revealed no intergroup differences. Further categorization into four biomarker-based cohorts demonstrated balanced baseline characteristics: Group A (high-IS + low-PIV, *n* = 43), Group B (high-IS + high-PIV, *n* = 38), Group C (low-IS + low-PIV, *n* = 70), and Group D (low-IS + high-PIV, *n* = 75; Tables 1–3).

**Table 1:** Correlation between baseline clinical characteristics and immunoscore in immunotherapy-treated patients

Variables	Total (*n* = 226)	Low IS (*n* = 145)	High IS (*n* = 81)	*P*
				
Age, M [IQR]	60.00 (54.00, 65.75)	60.00 (55.00, 64.00)	61.00 (54.00, 66.00)	0.901
Body Mass Index (BMI), M [IQR]	21.07 (17.99, 23.55)	21.14 (18.55, 23.34)	20.45 (17.32, 23.89)	0.528
Sex, *n* (%)				0.867
Female	46 (20.35)	30 (20.69)	16 (19.75)	
Male	180 (79.65)	115 (79.31)	65 (80.25)	
ASA grade, *n* (%)				0.320
II	199 (88.05)	130 (89.66)	69 (85.19)	
III	27 (11.95)	15 (10.34)	12 (14.81)	
Drugs type, *n* (%)				0.084
Pembrolizumab	125 (55.31)	74 (51.03)	51 (62.96)	
Others	101 (44.69)	71 (48.97)	30 (37.04)	
Neoadjuvant chemotherapy, *n* (%)				0.767
TP	200 (88.50)	129 (88.97)	71 (87.65)	
Others	26 (11.50)	16 (11.03)	10 (12.35)	
Pathological response, *n* (%)				**0.023**
Non-responders	126 (55.75)	89 (61.38)	37 (45.68)	
Responders	100 (44.25)	56 (38.62)	44 (54.32)	
Tumor location, n(%)				0.657
Upper third	20 (8.85)	14 (9.66)	6 (7.41)	
Middle third	128 (56.64)	79 (54.48)	49 (60.49)	
Lower third	78 (34.51)	52 (35.86)	26 (32.10)	
Drinking history, *n* (%)				0.972
No	98 (43.36)	63 (43.45)	35 (43.21)	
Yes	128 (56.64)	82 (56.55)	46 (56.79)	
Smoking history, *n* (%)				0.098
No	83 (36.73)	59 (40.69)	24 (29.63)	
Yes	143 (63.27)	86 (59.31)	57 (70.37)	
Hypertension, n(%)				0.207
No	170 (75.22)	113 (77.93)	57 (70.37)	
Yes	56 (24.78)	32 (22.07)	24 (29.63)	
Diabetes, *n*(%)				0.821
No	205 (90.71)	132 (91.03)	73 (90.12)	
Yes	21 (9.29)	13 (8.97)	8 (9.88)	
Coronary Heart Disease (CHD), *n* (%)				0.198
No	213 (94.25)	134 (92.41)	79 (97.53)	
Yes	13 (5.75)	11 (7.59)	2 (2.47)	
Chronic Obstructive Pulmonary Disease (COPD), *n* (%)				0.944
No	222 (98.23)	143 (98.62)	79 (97.53)	
Yes	4 (1.77)	2 (1.38)	2 (2.47)	
Clinical stage, *n* (%)				**0.028**
2	25 (11.06)	10 (6.90)	15 (18.52)	
3	183 (80.97)	123 (84.83)	60 (74.07)	
4a	18 (7.96)	12 (8.28)	6 (7.41)	

M, median; IQR, interquartile range

### Association of IS/PIV with pathological response

**Figure 3.**
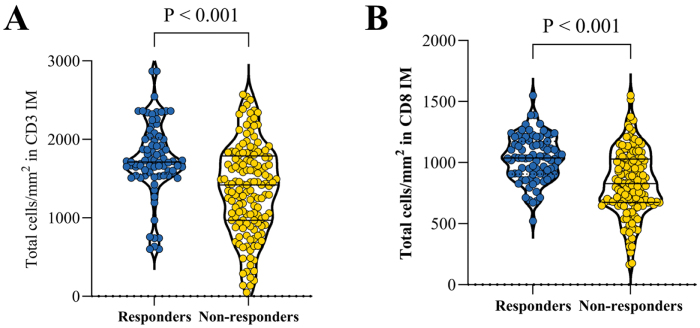
Scatter plot of total CD3^+^ and CD8^+^ cell density (cells/mm^2^) in the invasive margin (IM) between pathological responders and non-responders. Calculation method: CD3^+^/CD8^+^ cell density in IM = total lymphocyte count/IM total area (μm^2^).

**Figure 4.**
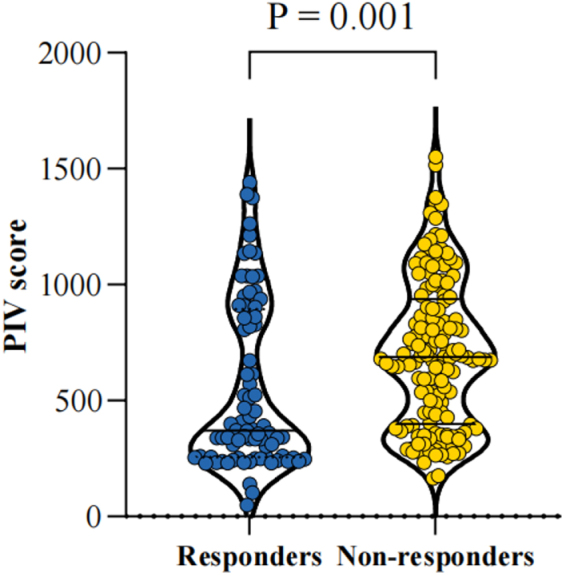
Scatter plot of pan-immune inflammation value in pathological responders vs. non-responders.

Postoperative assessment identified 100 (44.25%) pathological responders and 126 (55.75%) non-responders. Responders exhibited significantly higher median densities of CD3⁺ and CD8⁺ lymphocytes in the IM (1751 vs. 1367 and 1034 vs. 836 cells/mm^2^, respectively; Fig. 3) and a lower median PIV (554 vs. 705; Fig. 4) than non-responders (all *P* < 0.05). Univariate analysis confirmed higher response rates in high-IS versus low-IS (54.32% vs. 38.62%, *P* = 0.023; Table 1) and low-PIV versus high-PIV groups (67.26% vs. 21.24%, *P* < 0.001; Table 2). Combined analysis revealed differential response rates across groups (*P* = 0.028; Table 3): Group A (48.84%) and Group B (60.53%) exhibited superior responses to Group C (45.71%) and Group D (32.00%). Multivariable logistic regression adjusted for clinical stage (Table 4) demonstrated that Group D had significantly reduced odds of response versus Group A (OR = 0.41, 95% CI: 0.18–0.90, *P* = 0.027). Notably, stage IVa disease substantially increased non-response risk (OR = 7.97, 95% CI: 1.76–36.04, *P* = 0.007).

**Table 2:** Correlation between baseline clinical characteristics and peripheral immunovascular score (PIV) in immunotherapy-treated patients

Variables	High PIV (*n* = 113)	Low PIV (*n* = 113)	*P*
Age, M [IQR]	60.00 (54.00, 66.00)	59.00 (55.00, 65.00)	0.852
BMI, M [IQR]	20.86 (17.32, 23.89)	21.23 (18.73, 23.32)	0.433
Sex, *n* (%)			0.509
Female	21 (18.58)	25 (22.12)	
Male	92 (81.42)	88 (77.88)	
ASA grade, *n* (%)			0.305
II	97 (85.84)	102 (90.27)	
III	16 (14.16)	11 (9.73)	
Drugs type, *n* (%)			0.688
Pembrolizumab	49 (43.36)	52 (46.02)	
Others	64 (56.64)	61 (53.98)	
Neoadjuvant chemotherapy, *n* (%)			0.211
TP	10 (8.85)	16 (14.16)	
Others	103 (91.15)	97 (85.84)	
Pathological response, *n* (%)			**<0.001**
Non-responders	89 (78.76)	37 (32.74)	
Responders	24 (21.24)	76 (67.26)	
Tumor location, *n* (%)			0.406
Upper third	11 (9.73)	9 (7.96)	
Middle third	59 (52.21)	69 (61.06)	
Lower third	43 (38.05)	35 (30.97)	
Drinking history, *n* (%)			0.788
No	50 (44.25)	48 (42.48)	
Yes	63 (55.75)	65 (57.52)	
Smoking history, *n* (%)			0.890
No	42 (37.17)	41 (36.28)	
Yes	71 (62.83)	72 (63.72)	
Hypertension, *n* (%)			0.123
No	80 (70.80)	90 (79.65)	
Yes	33 (29.20)	23 (20.35)	
Diabetes, *n* (%)			0.492
No	101 (89.38)	104 (92.04)	
Yes	12 (10.62)	9 (7.96)	
CHD, *n* (%)			0.391
No	108 (95.58)	105 (92.92)	
Yes	5 (4.42)	8 (7.08)	
COPD, *n* (%)			1.000
No	111 (98.23)	111 (98.23)	
Yes	2 (1.77)	2 (1.77)	
Clinical stage, *n* (%)			0.133
2	13 (11.50)	12 (10.62)	
3	87 (76.99)	96 (84.96)	
4a	13 (11.50)	5 (4.42)	

M, median; IQR, interquartile range

**Table 3:** Correlation between baseline clinical characteristics and pan-immune inflammation value + immunoscore scores in immunotherapy-treated patients

Variables	Group A (*n* = 43)	Group B (*n* = 38)	Group C (*n* = 70)	Group D (*n* = 75)	*P*
Age, M (IQR)	63.00 (55.00,67.00)	57.00 (50.25,63.00)	58.00 (55.00,64.00)	60.00 (54.50,67.50)	0.108
BMI, M (IQR)	21.73 (18.97, 24.08)	19.73 (16.36, 22.73)	21.14 (19.08, 22.76)	21.14 (17.71, 24.15)	0.239
Sex, *n* (%)					
Female	9 (20.93)	7 (18.42)	16 (22.86)	14 (18.67)	
Male	34 (79.07)	31 (81.58)	54 (77.14)	61 (81.33)	
ASA grade, *n* (%)					0.723
II	36 (83.72)	33 (86.84)	62 (88.57)	68 (90.67)	
III	7 (16.28)	5 (13.16)	8 (11.43)	7 (9.33)	
Drugs type, *n* (%)					0.139
Pembrolizumab	16 (37.21)	14 (36.84)	39 (55.71)	32 (42.67)	
Others	27 (62.79)	24 (63.16)	31 (44.29)	43 (57.33)	
Neoadjuvant chemotherapy, *n* (%)					0.219
TP	7 (16.28)	3 (7.89)	11 (15.71)	5 (6.67)	
Others	36 (83.72)	35 (92.11)	59 (84.29)	70 (93.33)	
Pathological response, *n* (%)					**0.028**
Non-responders	22 (51.16)	15 (39.47)	38 (54.29)	51 (68.00)	
Responders	21 (48.84)	23 (60.53)	32 (45.71)	24 (32.00)	
Tumor location, *n* (%)					0.422
Upper third	3 (6.98)	3 (7.89)	6 (8.57)	8 (10.67)	
Middle third	30 (69.77)	19 (50.00)	42 (60.00)	37 (49.33)	
Lower third	10 (23.26)	16 (42.11)	22 (31.43)	30 (40.00)	
Drinking history, *n* (%)					0.752
No	21 (48.84)	14 (36.84)	30 (42.86)	33 (44.00)	
Yes	22 (51.16)	24 (63.16)	40 (57.14)	42 (56.00)	
Smoking history, *n* (%)					0.343
No	14 (32.56)	10 (26.32)	27 (38.57)	32 (42.67)	
Yes	29 (67.44)	28 (73.68)	43 (61.43)	43 (57.33)	
Hypertension, *n* (%)					0.553
No	31 (72.09)	26 (68.42)	53 (75.71)	60 (80.00)	
Yes	12 (27.91)	12 (31.58)	17 (24.29)	15 (20.00)	
CHD, *n* (%)					0.157
No	43 (100.00)	36 (94.74)	63 (90.00)	71 (94.67)	
Yes	0 (0.00)	2 (5.26)	7 (10.00)	4 (5.33)	
COPD, *n* (%)					0.080
No	43 (100.00)	36 (94.74)	68 (97.14)	75 (100.00)	
Yes	0 (0.00)	2 (5.26)	2 (2.86)	0 (0.00)	
Clinical stage, *n* (%)					0.201
2	10 (23.26)	5 (13.16)	5 (7.14)	5 (6.67)	
3	30 (69.77)	30 (78.95)	59 (84.29)	64 (85.33)	
4a	3 (6.98)	3 (7.89)	6 (8.57)	6 (8.00)	

Group A: high IS + low PIV; Group B: high IS + high PIV; Group C: low IS + low PIV; and Group D: low IS + high PIV.

**Table 4:** Univariate and multivariate logistic regression analysis of pathological response

Variables	Comparisons	Univariate regression analysis		Multivariate regression analysis	
		OR (95%CI)	*P*	OR (95%CI)	*P*
IS + PIV group	A vs. B	0.92 (0.38–2.22)	0.850	0.85 (0.34–2.10)	0.721
	A vs. C	1.68 (0.78–3.62)	0.182	1.52 (0.69–3.36)	0.300
	A vs. D	2.68 (1.24–5.82)	**0.012**	2.47 (1.11–5.48)	**0.027**
Age		1.00 (0.98–1.03)	0.869		
Sex	Female vs. male	1.07 (0.56–2.06)	0.830		
BMI		1.01 (0.95–1.07)	0.832		
Drinking	No vs. yes	1.05 (0.62–1.78)	0.863		
Smoking	No vs. yes	1.01 (0.59–1.71)	0.983		
Hypertension	No vs. yes	0.92 (0.52–1.62)	0.766		
Diabetes	No vs. yes	1.32 (0.53–3.33)	0.552		
CHD	No vs. yes	1.29 (0.41–4.07)	0.666		
Location	Upper vs. middle	0.47 (0.17–1.30)	0.147		
	Upper vs. lower	0.58 (0.20–1.68)	0.319		
ASA	II vs. III.	1.18 (0.52–2.66)	0.696		
Drug	Pembrolizumab vs. others	0.66 (0.39–1.12)	0.126		
Chemotherapy	TP vs. others	1.09 (0.48–2.48)	0.835		
Clinical stage	2 vs. 3	2.24 (0.94–5.33)	0.069	1.90 (0.77–4.65)	0.162
	2 vs. 4a	8.89 (2.01–39.22)	**0.004**	7.97 (1.76–36.04)	**0.007**

### Ability of IS and PIV to discriminate pathological responders

**Figure 5.**
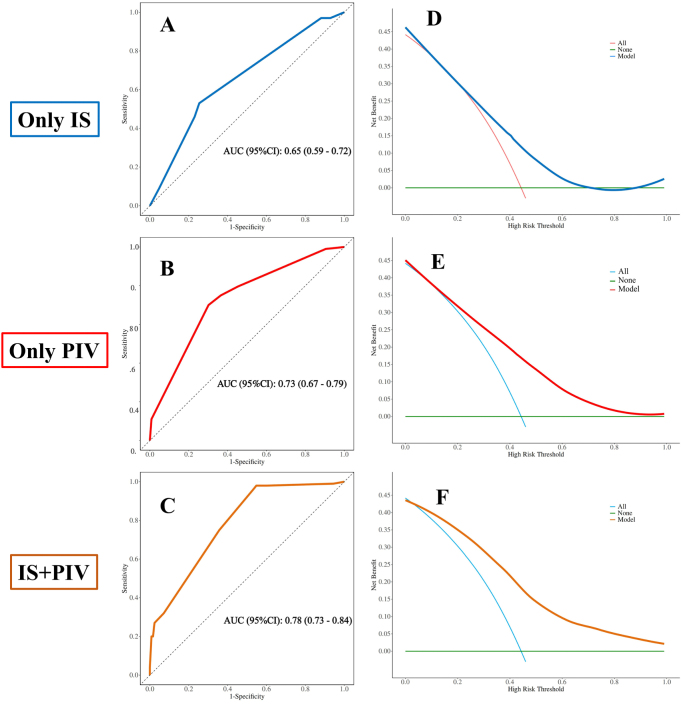
Discriminatory performance of clinical stage combined with immunoscore (IS), pan-immune inflammation value (PIV), and their combination for pathological response. (A) ROC curve of clinical stage + IS [area under the curve (AUC) 0.65, 95% confidence interval (CI) 0.59–0.72]. (B) ROC curve of clinical stage + PIV (AUC 0.73, 95% CI 0.67–0.79). (C) ROC curve of clinical stage + IS + PIV (AUC 0.78, 95% CI 0.73–0.84). (D–F) Decision curve analysis of (D) clinical stage + IS, (E) clinical stage + PIV, and (F) clinical stage + IS + PIV models.

ROC analysis (Fig. 5) showed areas under the curve (AUCs) of 0.65 (95% CI: 0.59–0.72) for clinical stage + IS, 0.73 (95% CI: 0.67–0.79) for clinical stage + PIV, and 0.78 (95% CI: 0.73–0.84) for the triple-parameter model (stage + IS + PIV) in discriminating pathological responders. Decision curve analysis (DCA) confirmed superior net clinical benefit of the combined model across threshold probabilities of 0.1–0.8 (Fig. 5). This discriminatory utility was preserved in subgroup analyses confined to Stage II + III, Stage III-only, and Stage II-only patients, with detailed performance metrics provided in the Supplementary Material (see Supplemental Digital Content Figure S1, available at http://links.lww.com/JS9/F253).

### Association of IS/PIV with survival outcomes

**Figure 6.**
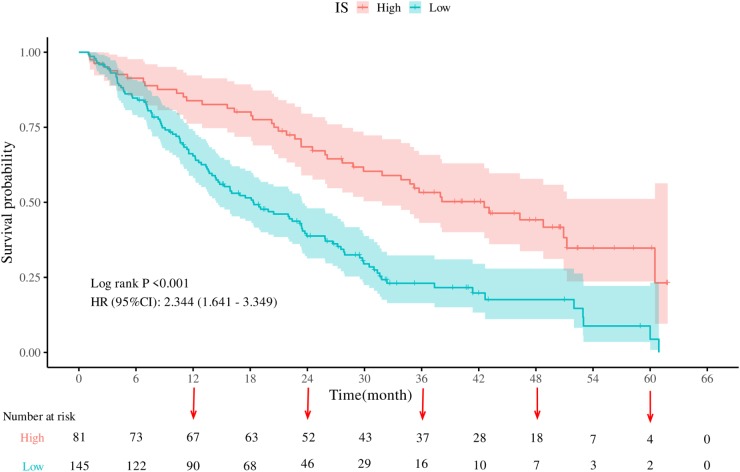
Kaplan–Meier survival curves of overall survival (OS) in IS.

**Figure 7.**
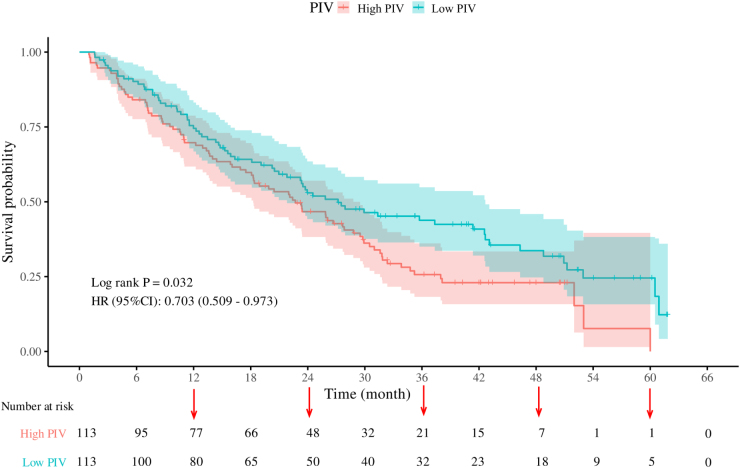
Kaplan–Meier survival curves of overall survival (OS) in pan-immune inflammation value.

**Figure 8.**
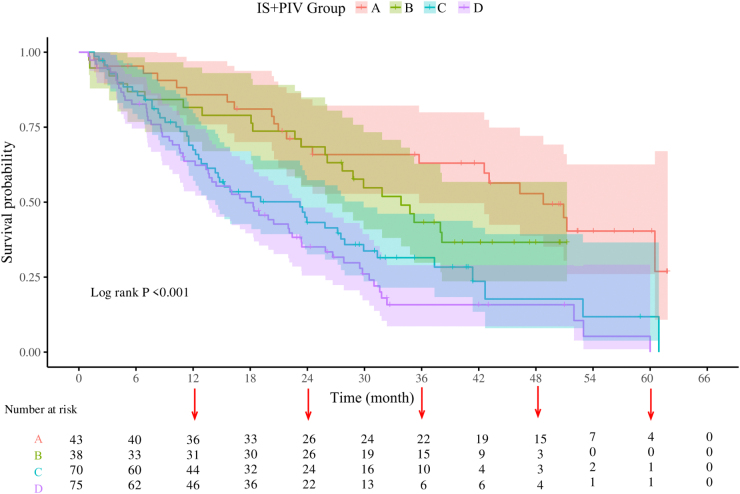
Kaplan–Meier survival curves of overall survival (OS) in pan-immune inflammation value + immunoscore.

Kaplan-Meier curves revealed significantly prolonged OS in high-IS versus low-IS (HR = 2.344, 95% CI: 1.641–3.349, *P* < 0.001; Fig. 6) and low-PIV versus high-PIV groups (HR = 1.51, 95% CI: 1.036–2.201, *P* = 0.032; Fig. 7). Striking survival differences emerged across combined groups (*P* < 0.001; Fig. 8): Group A (high-IS + low-PIV) had optimal survival, followed by Group B (high-IS + high-PIV) and Group C (low-IS + low-PIV), while Group D (low-IS + high-PIV) demonstrated worst prognosis. Multivariable Cox regression (Table 5) showed that Group D had 2.85-fold increased mortality risk versus Group A (HR = 2.85, 95% CI: 1.70–4.77, *P* < 0.001), with Group C at 2.19-fold risk (HR = 2.19, 95% CI: 1.30–3.68, *P* = 0.003); Group B showed no significant difference (HR = 1.59, 95% CI: 0.87–2.93, *P* = 0.134). Stage IVa conferred extreme mortality risk (HR = 111.49, 95% CI: 40.46–307.23, *P* < 0.001). Notably, the significant survival discrimination among the four IS + PIV groups was consistently observed across all subgroups, including Stage II + III, Stage III-only, and Stage II-only patients (all log-rank *P* < 0.05) (Supplemental Digital Content Figure S2, available at http://links.lww.com/JS9/F253).

**Table 5:** Uni- and multivariate analysis of overall survival

Variables	Comparisons	Univariate Cox regression analysis		Multivariate Cox regression analysis	
		OR (95% CI)	*P*	OR (95% CI)	*P*
IS + PIV group	A vs. B	1.53 (0.84–2.77)	0.163	1.59 (0.87–2.93)	0.134
	A vs. C	2.51 (1.50–4.20)	<0.001	2.19 (1.30–3.68)	0.003
	A vs. D	3.25 (1.96–5.37)	<0.001	2.85 (1.70–4.77)	<.001
Age		1.01 (0.99–1.03)	0.259		
Sex	Female vs. male	0.80 (0.55–1.18)	0.258		
BMI		1.03 (1.00–1.07)	0.080		
Drinking	No vs. yes	0.99 (0.72–1.37)	0.964		
Smoking	No vs. yes	0.85 (0.61–1.18)	0.343		
Hypertension	No vs. yes	1.03 (0.71–1.49)	0.887		
Diabetes	No vs. yes	0.89 (0.49–1.60)	0.692		
CHD	No vs. yes	0.77 (0.38–1.57)	0.472		
COPD	No vs. yes	0.40 (0.10–1.65)	0.206		
Location of tumor	Upper vs. middle	0.95 (0.52–1.73)	0.855		
	Upper vs. lower	0.94 (0.50–1.77)	0.854		
ASA	II vs. III.	0.57 (0.34–0.98)	0.043	0.70 (0.40–1.20)	0.191
Drugs type	Pembrolizumab vs. others	0.82 (0.60–1.13)	0.221		
Neoadjuvant chemotherapy	TP vs. others	1.20 (0.73–1.97)	0.463		
Pathological response	Non-responders vs. responders	0.57 (0.41–0.79)	<0.001	0.78 (0.55–1.11)	0.166
Clinical stage	2 vs. 3	6.19 (2.81–13.65)	<0.001	4.66 (2.10–10.33)	<0.001
	2 vs. 4a	130.19 (48.26–351.21)	<0.001	111.49 (40.46–307.23)	<0.001

### Predictive performance of IS/PIV models for survival

**Figure 9.**
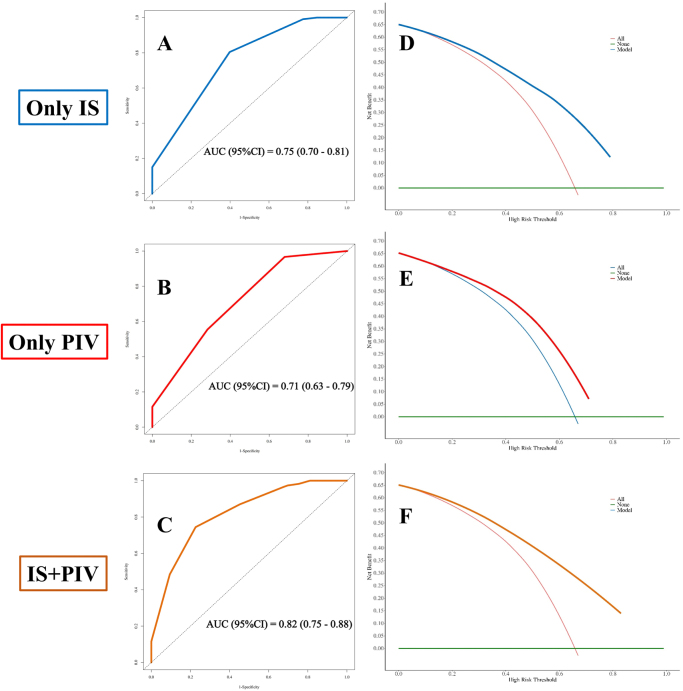
Prognostic performance of clinical stage combined with immunoscore (IS), pan-immune inflammation value (PIV), and their combination for 36-month survival. (A) ROC curve of clinical stage + IS [area under the curve (AUC) 0.75, 95% confidence interval (CI) 0.70–0.81]. (B) ROC curve of clinical stage + PIV (AUC 0.71, 95% CI 0.63–0.79). (C) ROC curve of clinical stage + IS + PIV (AUC 0.82, 95% CI 0.75–0.88). (D–F) Decision curve analysis of (D) clinical stage + IS, (E) clinical stage + PIV, and (F) clinical stage + IS + PIV models.

The combined model Receiver Operating Characteristic (ROC) analysis (Fig. 9) yielded AUCs of 0.75 (95% CI: 0.70–0.81) for stage + IS, 0.71 (95% CI: 0.63–0.79) for stage + PIV, and 0.82 (95% CI: 0.75–0.88) for the triple-predictor model at 36 months. DCA reaffirmed greater net benefit of the integrated model across clinical decision thresholds (Fig. 9D–F). Similarly, the model maintained robust predictive accuracy for 36-month OS in analyses of the Stage II + III, Stage III-only, and Stage II-only subgroups (see Supplemental Digital Content Figure S3, available at http://links.lww.com/JS9/F253).

## Discussion

This study pioneers and validates an innovative dual-dimensional assessment strategy integrating local tumor immune microenvironment status (quantified by IS) and systemic inflammatory burden (reflected by PIV) to assess pathological response and predict long-term survival in locally advanced ESCC patients receiving NACI. The combined IS + PIV model demonstrated superior discriminatory and predictive performance, achieving an AUC of 0.78 for major pathological response and 0.82 for OS – significantly outperforming individual biomarkers (IS, PIV) or clinical staging. Crucially, this model enabled refined risk stratification: Group A (high-IS + low-PIV) represented optimal responders with a 48.8% pathological response rate and the most favorable survival outcomes, while Group D (low-IS + high-PIV) constituted a high-risk subpopulation exhibiting significantly reduced pathological response and a 2.85-fold increased mortality risk versus Group A (HR = 2.85, 95% CI: 1.70–4.77; *P* < 0.001). The model’s clinical utility is underscored by its operational feasibility – IS derivation relies on routine CD3/CD8 IHC, and PIV calculation requires only standard complete blood count (CBC) parameters (neutrophils, lymphocytes, monocytes, and platelets) – rendering both components cost-effective and widely accessible in clinical practice^[8,9]^.

Precision identification of ESCC patients who may benefit from NACI remains a critical clinical challenge. Previous studies have independently established the value of local immune infiltration (e.g., CD3^+^/CD8^+^ T-cell density assessed via IS) and systemic inflammation (e.g., PIV) in predicting response to immune checkpoint inhibitors (ICIs)^[10]^. Across multiple solid tumors – including melanoma, breast cancer, and colorectal cancer – tumor-infiltrating lymphocyte (TIL) density has been recognized as a key biomarker for immunotherapy and is being progressively incorporated into clinical practice guidelines^[11,12]^. In esophageal cancer specifically, elevated CD3^+^ T-cell density in pretreatment biopsies correlates with improved pathological response rates following neoadjuvant chemotherapy^[13]^. Concurrently, multiple studies consistently demonstrate that baseline elevation of PIV associates with primary resistance to ICIs, significantly reduced objective response rates, and shortened progression-free survival (PFS) across diverse malignancies^[14]^. For instance, in non-small cell lung cancer, PIV serves as a robust prognostic indicator capable of reliably predicting both OS and PFS^[15]^. Nevertheless, single-dimensional biomarkers – including isolated PD-L1 expression, tumor mutational burden, local TIL density, or systemic inflammatory indices – often exhibit unstable predictive performance with inherent limitations^[16]^. This primarily stems from the biological complexity of antitumor immune responses: a dynamic continuum involving bidirectional interplay between local tumor microenvironments and systemic immunity. Isolated assessment of local immune infiltration risks overlooking the potent immunosuppressive effects of systemic inflammation on infiltrating T-cell function, whereas exclusive focus on systemic inflammation fails to capture spatial immune activity within tumors.

The refined risk stratification also revealed a nuanced profile in Group B (high IS + high PIV), which exhibited a considerable pathological response rate (60.53%) yet without a consequent significant survival benefit compared with Group A (HR = 1.59, *P* = 0.134). This observation suggests that a high systemic inflammatory burden (as indicated by high PIV) may ultimately impede the translation of an initial robust pathological response into a long-term survival advantage, potentially through promoting a pro-tumorigenic microenvironment that fosters recurrence. The limited sample size of Group B (*n* = 38) may also contribute to the lack of statistical power to detect a significant difference in survival.

The breakthrough significance of our findings lies in revealing that the synergy between local immune activation and a systemic low-inflammatory state constitutes the key biological basis underlying NACI efficacy. Specifically, the favorable prognosis observed in the patient subgroup (Group A) characterized by the “high IS + low PIV” profile is underpinned by profound immunological mechanisms. A high IS signifies the effective enrichment of functionally active CD8^+^ T cells within the tumor margin (IM). These cells express high levels of PD-1^[17]^, representing the direct targets of PD-1 inhibitors. PD-1 blockade significantly activates the cytotoxic function of these CD8⁺ T cells^[18]^, thereby driving effective tumor cell clearance. This mechanism explains the significantly improved pathological response rate observed in the high IS group in this study (54.3% vs. 38.6%, *P* = 0.023). Conversely, a low PIV indicates a relatively low systemic inflammatory burden. This low-inflammatory environment is crucial for maintaining T cell effector function. Studies demonstrate that a low PIV may effectively mitigate the suppression and exhaustion of tumor-infiltrating T cells, potentially through inhibiting the activation of key pro-inflammatory pathways such as IL-6/JAK/STAT3, which, as reported by Tsukamoto *et al*, can induce PD-L1 upregulation on myeloid cells^[19]^. Therefore, the “high IS + low PIV” profile represents an ideal immune state characterized by “robust local immune effector function” coupled with “alleviated systemic immunosuppression,” which establishes a positive feedback loop. Effective local tumor killing releases tumor antigens, initiating and amplifying systemic anti-tumor immune responses. This process maintains or reduces systemic inflammation, which in turn sustains and enhances the function of local infiltrating T cells^[20,21]^. Conversely, the loss of survival benefit observed in the “high IS + high PIV” group (Group B; HR = 1.59, *P* = 0.134) stems precisely from the counteracting effect of high systemic inflammation. Mechanistically, an elevated PIV reflects a pro-tumorigenic systemic environment that is frequently driven by the activation of pro-inflammatory signaling pathways, most notably IL-6/JAK/STAT3^[22]^. This activation can directly suppress the cytotoxic function of CD8^+^ T cells and promote the differentiation of myeloid-derived suppressor cells^[23]^. Furthermore, as demonstrated by Tsukamoto *et al*, IL-6/STAT3 signaling can induce the upregulation of PD-L1 on myeloid cells within the tumor microenvironment^[19]^. This creates an adaptive immune resistance mechanism that effectively neutralizes the local anti-tumor activity of PD-1 inhibitor-targeted CD8^+^ T cells, ultimately leading to T cell exhaustion and diminished immunotherapy benefit, even in the context of a high IS.

The superiority of this combined assessment based on local and systemic immune states finds strong corroboration in pan-cancer studies. For instance, patients with melanoma responding to PD-1 inhibitors typically exhibit both a high T-cell-inflamed gene expression profile and low serum IL-8 levels^[24]^. In gastric cancer neoadjuvant therapy, patients achieving a major pathological response similarly demonstrate high intratumoral CD8⁺ T-cell density coupled with a low neutrophil-to-lymphocyte ratio^[25]^. Furthermore, pivotal clinical trials in esophageal cancer confirmed that the survival benefit from adjuvant nivolumab predominantly accrued to the subgroup characterized by high TILs and low C-reactive protein levels^[26,27]^. The IS + PIV combined model developed in this study (AUC = 0.82) leverages its ability to precisely capture this transcancer immunological unity. This not only provides a robust predictive tool for NACI efficacy and prognosis in ESCC patients but also offers critical insights into the potential mechanisms underlying immunotherapy resistance in this malignancy – particularly for the high-risk “low IS + high PIV” subgroup, which exhibits an exceptionally elevated mortality risk.

Given that both IS and PIV derive from routine clinical tests, the translational potential of this combined model is substantial and clinically feasible. Technically, IS assessment can be standardized using automated image analysis software applied to CD3/CD8-stained sections from readily available FFPE specimens. PIV calculation, conversely, relies solely on four parameters extracted from standard preoperative CBC reports, requiring no specialized assays or additional costs. This reliance on ubiquitous clinical data sources underpins the model’s broader applicability across diverse healthcare settings.

### Limitations

This study, based on a single-center retrospective design, requires validation of the model’s generalizability in multicenter prospective cohorts. The absence of dynamic monitoring of IS/PIV changes pre- and post-treatment limits its utility for response assessment. While PIV serves as a composite inflammatory indicator, it does not delineate the individual contributions of specific immune cell subsets or inflammatory cytokines. Furthermore, the current IS scoring methodology relies on manual hotspot selection; future efforts should focus on implementing whole-slide digital automated analysis to enhance standardization. Finally, as all patients received PD-1 inhibitors, the applicability of our IS + PIV model to other immunotherapies, such as CTLA-4 inhibitors, requires future validation.

## Conclusion

This study provides the first evidence that combined assessment of the local tumor IS and the PIV significantly outperforms single biomarkers and clinical staging, being accurate both in assessing pathological response and in predicting long-term survival outcomes (OS) following NACI in locally advanced ESCC. This dual-parameter model offers an efficient and cost-effective tool for post-treatment prognostication and stratification.

## Data Availability

The datasets used and analyzed during the current study are available from the corresponding author on reasonable request.

## Research registration unique identifying number (UIN)

This retrospective study has been registered with the Chinese Clinical Trial Registry (ChiCTR; Registration Number: ChiCTR2400082743). According to the guidelines of the International Committee of Medical Journal Editors (ICMJE), retrospective studies may be registered prior to the analysis of results, and the timing of registration for this study complies with the requirements.
